# Feasibility of Multiple Examinations Using ^68^Ga-Labelled Collagelin Analogues: Organ Distribution in Rat for Extrapolation to Human Organ and Whole-Body Radiation Dosimetry

**DOI:** 10.3390/ph9020031

**Published:** 2016-06-06

**Authors:** Irina Velikyan, Ulrika Rosenström, Thomas N. Bulenga, Olof Eriksson, Gunnar Antoni

**Affiliations:** Department of Medicinal Chemistry, Uppsala University, SE-75183 Uppsala, Sweden; ulrika.rosenstrom@orgfarm.uu.se (U.R.); tomndaro@yahoo.com (T.N.B.); olof.eriksson@pet.medchem.uu.se (O.E.); gunnar.antoni@akademiska.se (G.A.)

**Keywords:** gallium-68, collagelin, dosimetry, fibrosis, imaging

## Abstract

*Objectives*: Fibrosis is involved in many chronic diseases. It affects the functionality of vital organs, such as liver, lung, heart and kidney. Two novel imaging agents for positron emission tomography (PET) imaging of fibrosis have previously pre-clinically demonstrated promising target binding and organ distribution characteristics. However, the relevant disease monitoring in the clinical setup would require multiple repetitive examinations per year. Thus, it is of paramount importance to investigate the absorbed doses and total effective doses and thus, the potential maximum number of examinations per year. *Methods*: Two cyclic peptide (c[CPGRVMHGLHLGDDEGPC]) analogues coupled via an ethylene glycol linker (EG_2_) to either 2-(4,7-bis(2-(*tert*-butoxy)-2-oxoethyl)-1,4,7-triazonan-1-yl)acetic acid (NO2A-Col) or 4-(4,7-bis(2-(*tert*-butoxy)-2-oxoethyl)-1,4,7-triazacyclononan-1-yl)-5-(tert-butoxy)-5-oxopentanoic acid (NODAGA-Col) were labelled with ^68^Ga. The resulting agents, [^68^Ga]Ga-NO2A-Col and [^68^Ga]Ga-NODAGA-Col, were administered in the tail vein of male and female Sprague–Dawley rats (*N* = 24). An *ex vivo* organ distribution study was performed at the 5-, 10-, 20-, 40-, 60- and 120-min time points. The resulting data were extrapolated for the estimation of human organ and total body absorbed and total effective doses using Organ Level Internal Dose Assessment Code software (OLINDA/EXM 1.1) assuming a similar organ distribution pattern between the species. Time-integrated radioactivity in each organ was calculated by trapezoidal integration followed by a single-exponential fit to the data points extrapolated to infinity. The resulting values were used for the residence time calculation. *Results*: *Ex vivo* organ distribution data revealed fast blood clearance and washout from most of the organs. Although the highest organ absorbed dose was found for kidneys (0.1 mGy/MBq), this organ was not the dose-limiting one and would allow for the administration of over 1460 MBq per year for both [^68^Ga]Ga-NO2A-Col and [^68^Ga]Ga-NODAGA-Col. The total effective dose was the limiting parameter with 0.0155/0.0156 (female/male) mSv/MBq and 0.0164/0.0158 (female/male) mSv/MBq, respectively, for [^68^Ga]Ga-NO2A-Col and [^68^Ga]Ga-NODAGA-Col. This corresponded to the total amount of radioactivity that could be administered per year of 643 and 621 MBq before reaching the annual limit of 10 mSv. Thus, up to six examinations would be possible. The residence time and organ absorbed doses in liver and spleen were higher for [^68^Ga]Ga-NODAGA-Col as compared to [^68^Ga]Ga-NO2A-Col. *Conclusion*: The limiting parameter for the administered dose was the total effective dose that would allow for at least six examinations per year that might be sufficient for adequate disease monitoring in longitudinal studies and a routine clinical setup.

## 1. Introduction

The consequences of fibrosis affecting the functionality of vital organs, such as liver, lung, heart and kidney, can be lethal, and it is crucial to diagnose the disorder in the early stage of its development, as well as to predict the disease progression and response to therapy. The feasibility of the development of a non-invasive, early, specific, sensitive and quantitative diagnostic method of fibrosis at the molecular level has been explored previously [1,2]. The underlying hypothesis was the specific affinity of collagelin ligands to collagen I and III that excessively accumulate in tissue causing elasticity loss. Such ligands labelled with positron emitting ^68^Ga (t_1/2_= 68 min, 89% β^+^) in combination with positron emission tomography-computed tomography (PET-CT) would allow accurate and direct quantitative assessment of the status of fibrosis for the determination of the disease progression and response to treatment. A single examination using this technique results in a whole body scan with accurate and specific localization of the target and real-time evaluation of physiology and pathology. PET-CT would allow one to overcome the non-specificity and insufficient sensitivity of non-invasive anatomical imaging techniques, such as ultrasound, transient elastography (TE), ultrasound Doppler technology, acoustic radiation force impulse imaging, magnetic resonance imaging (MRI) and computed tomography (CT). It would also allow one to avoid invasive diagnostic methods that can be used only in the late stage of the fibrotic process and that are associated with tissue sampling error and side effects, such as infection, hemorrhage and patient discomfort. Moreover, multiple biopsies for the determination of disease progression and treatment response monitoring are rarely possible in clinical practice.

The choice of the ^68^Ga radionuclide was justified by its advantageous characteristics and application versatility [3,4,5,6,7]. In particular, its availability from a generator system and labelling techniques amenable both to manual radiopharmacy practice and automated manufacturing would make the respective imaging agent available and affordable at any distant clinical center, providing worldwide accessibility.

As mentioned above, the potential for the imaging of fibrosis using two collagelin analogues comprising different derivatives of 1,4,7-triazacyclononane-*N*,*N*′,*N*″-triacetic acid (NOTA), [^68^Ga]Ga-NO2A-Col (2-(4,7-bis(2-(*tert*-butoxy)-2-oxoethyl)-1,4,7-triazonan-1-yl)acetic acid (NO2A-Col)) and [^68^Ga]Ga-NODAGA-Col (4-(4,7-bis(2-(*tert*-butoxy)-2-oxoethyl)-1,4,7-triazacyclononan-1-yl)-5-(*tert*-butoxy)-5-oxopentanoic acid (NODAGA-Col)), has been demonstrated previously [2]. In particular, both tracers were cleared quickly from blood and normal tissue after intravenous administration to healthy rats, and the binding specificity was confirmed *in vitro* in cryosections of dog left ventricle myocardium with histologically-confirmed fibrosis. The results were encouraging; however, the development and evaluation of a new radiopharmaceutical are a major undertaking, which requires considerable finance and resources expenses. Another aspect is the ethics of animal usage and the necessity to decrease the number of sacrificed animals. It is therefore rational to first investigate the most critical factors influencing the decision of initiating such a study. The adequate disease monitoring in the clinical setup would require multiple repetitive examinations per year and longitudinal studies. Thus, it was of paramount importance to conduct a dosimetry study and to estimate the absorbed doses and total effective doses and, thus, the potential maximum number of examinations per year.

Dosimetry is a crucial part of nuclear medicine required for the assessment of potential radiotoxicity to the essential radiosensitive organs, such as bone marrow, organs with a physiological uptake of the radiopharmaceutical and healthy tissue surrounding lesions and excretory organs [8,9]. Thus, dosimetry evaluates the distribution and kinetics of an administered radiopharmaceutical [10]. A high radiation dose to vital organs may prevent the use of an imaging agent. The dosimetry investigation can reveal at the very early stage of the development if the tracer presents practical interest or must be modified in order to provide the desired biodistribution and pharmacokinetics. Thus, it is of utmost importance to investigate dosimetry before undertaking expensive and resource-consuming studies involving model animals. Moreover, such an approach would decrease the unnecessary consumption of animals, thus meeting ethical considerations. Radiotoxicity to healthy tissue/organs must be minimized, and the respective administered radioactivity dose can be restricted by the absorbed dose to normal organs, which must be kept within the maximum tolerated dose for the organs at risk, such as bone marrow and kidneys. In the case of peptide-based agents, the kidney is often a limiting organ. The total effective dose can present restrictions, as well.

This study reports on the dosimetry of two collagelin analogues, [^68^Ga]Ga-NO2A-Col and [^68^Ga]Ga-NODAGA-Col [2], extrapolated to human from rat biodistribution data in order to determine the potential of the tracers for annual multiple PET-CT examinations.

## 2. Material and Methods

### 2.1. Materials

The purchased chemicals were used without further purification: sodium acetate buffer (pH 4.6, 31048, Sigma-Aldrich, Stockholm, Sweden), 30% HCl (Ultrapure, 1.00318.0250 Merck, Sigma-Aldrich) and trifluoroacetic acid (TFA, Merck, Darmstadt, Germany). Deionized water (18.2 MΩ), produced with a Purelab Maxima Elga system (Bucks, UK), was used in ^68^Ga-labelling reactions.

### 2.2. Peptide Synthesis and Radiochemistry

The solid-phase peptide synthesis of the cyclic peptide c[CPGRVMHGLHLGDDEGPC] and the analogues conjugated to 2-(4,7-bis(2-(*tert*-butoxy)-2-oxoethyl)-1,4,7-triazonan-1-yl)acetic acid (NOTA(tBu)_2_) or to 4-(4,7-bis(2-(*tert*-butoxy)-2-oxoethyl)-1,4,7-triazacyclononan-1-yl)-5-(*tert*-butoxy)-5-oxopentanoic acid (NODAGA(tBu)_3_ via an ethylene glycol link (EG_2_) was reported previously [2]. The resulting precursors, NOTA-EG_2_-c[CPGRVMHGLHLGDDEGPC] (NO2A-Col, 23.2 µg) and NODAGA-EG_2_-c[CPGRVMHGLHLGDDEGPC] (NODAGA-Col, 23.9 µg), were labelled with ^68^Ga available from a ^68^Ge/^68^Ga generator system (1850 MBq, IGG100-50, Eckert & Ziegler, Eurotope GmbH, Berlin, Germany), as described previously [2].

### 2.3. Organ Distribution Study

Animal studies were performed using healthy female and male Sprague–Dawley rats: one male and one female animal per time point and tracer (Table 1). The animal experiments were approved by the local Ethics Committee for Animal Research and performed in accordance with local institutional and Swedish national rules and regulations. The animals were kept at a constant temperature (20 °C) and humidity (50%) in a 12-h light-dark cycle and given free access to food and water.

[^68^Ga]Ga-NO2A-Col or [^68^Ga]Ga-NODAGA-Col was injected into the tail vein of rats as a bolus with 500 µL phosphate-buffered saline (pH 7.4) as the vehicle (Table 1). The injected amount of the peptides was 0.8 ± 0.2 nanomoles. The animals were euthanized by a CO_2_–O_2_ mixture at 5, 10, 20, 40, 60 and 120 min post-injection. The radioactivity of the excised organs was measured in a well-type NaI(Tl) scintillation counter, applying correction for dead-time and radioactivity decay. The weight of the tissues was determined simultaneously. The remaining carcass was also measured in order to monitor the radioactivity elimination and recovery. Samples from blood, heart, lung, liver, spleen, adrenal glands, kidneys, intestines, with or without contents, muscle, testis, bone, brain, pancreas, urine bladder and bone marrow were collected. The radioactivity readings were decay-corrected to the time of the injection, and the results were expressed as standardized uptake values (SUV; Equation (1)), where the radioactivity of an organ and injected radioactivity was expressed in MBq, and the weight of a rat and organ was expressed in g.

(1) $$SUV=\frac{Radioactivity_{organ}\times weight_{rat}}{Radioactivity_{injected}\times weight_{organ}}$$

### 2.4. Dosimetric Calculations

Human organ and total body absorbed and effective dose estimates were calculated according to the Medical Internal Radionuclide Dose (MIRD) procedure using the measured residence times and the dose rate S-values [11,12]. The SUV values in rat were first multiplied with the appropriate radioactivity decay factor dependent on the time point of the data post-injection, then multiplied by standard organ masses and divided by the standard total-body weight for the standard adult man phantom obtained from the OLINDA/EXM 1.1 software (Organ Level Internal Dose Assessment Code, Vanderbilt University, Nashville, TN, USA, 2007) [13]. The values obtained correspond to the fraction of injected radioactivity (%IA) per organ in man as a function of time (Equation (2), where *SUV_A_* is the SUVs of rat organs; *g_organ_* and *kg_TBweight_* are respectively the standard organ weight and standard total body weight of a human).

(2) $${\left(\%IA_{Organ}\right)}_{human}=SUV_{A}\times {\left(\frac{g_{organ}}{kg_{TBweight}}\right)}_{human}$$

The cumulative radioactivity for the organs was determined by integrating the area under the time-radioactivity curves for each organ using trapezoidal approximation of the collected kinetic data, followed by extrapolation of remaining points from the last time point to infinity by a single exponential fit. The residence time for each organ is equal to the number of disintegrations (MBq × h/MBq). Bone marrow residence time was assessed according to the bone marrow blood model [14]. The residence time for the remaining carcass was calculated in the same way as for the other organs, and this value was used as input in the OLINDA software as “remainder”. The “remainder” of the body residence time was calculated from SUV due to radioactivity measured in the rest of the body after the removal of the source organs and tail. The difference between the theoretical residence time and the calculated residence time of the source organs plus the remainder of the body was assumed to have been in the tail or excreted. The kidney absorbed dose was calculated based on a multi-region kidney model phantom [15]. The total target organ absorbed dose (*D*) is contributed by all source organs and is expressed by Equation (3), where Ã is the time-integrated radioactivity in the source tissue (r_S_) and *S* is the radionuclide-specific dose rate values [11]:

(3) $$D\left(r_{T},t\right)=\underset{r_{S}}{\sum }Ã\left(r_{S},t\right)\times S\left(r_{T}\leftarrow r_{S},t\right)$$

### 2.5. Statistical Analysis

Non-linear regression analyses were made with GraphPad Prism software (Version 5.02 for Windows, GraphPad Software Inc., La Jolla, CA, USA), and the goodness of fit to the variables was presented as R^2^ values.

## 3. Results

### 3.1. Radiochemistry

**Figure 1:**
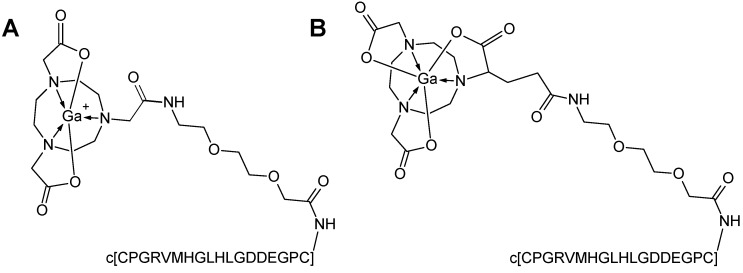
Chemical formulae of [^68^Ga]Ga-NO2A-Col with a positively-charged complex moiety (**A**) and [^68^Ga]Ga-NODAGA-Col with a neutral complex moiety (**B**), where c[CPGRVMHGLHLGDDEGPC] is a cyclic peptide, collagelin, coupled to the chelator moiety via an ethylene glycol linker (EG_2_).

Two cyclic peptide (c[CPGRVMHGLHLGDDEGPC]) analogues coupled via an ethylene glycol linker (EG_2_) to either 2-(4,7-bis(2-(*tert*-butoxy)-2-oxoethyl)-1,4,7-triazonan-1-yl)acetic acid (NO2A-Col) or 4-(4,7-bis(2-(*tert*-butoxy)-2-oxoethyl)-1,4,7-triazacyclononan-1-yl)-5-(tert-butoxy)-5-oxopentanoic acid (NODAGA-Col) were labelled with ^68^Ga. The non-decay-corrected radiochemical yield was 82% ± 6% with a radiochemical purity of 95% ± 3%. The effective specific radioactivity was 50 ± 10 MBq/nmol (Figure 1).

### 3.2. Organ Distribution and Kinetics

**Figure 2:**
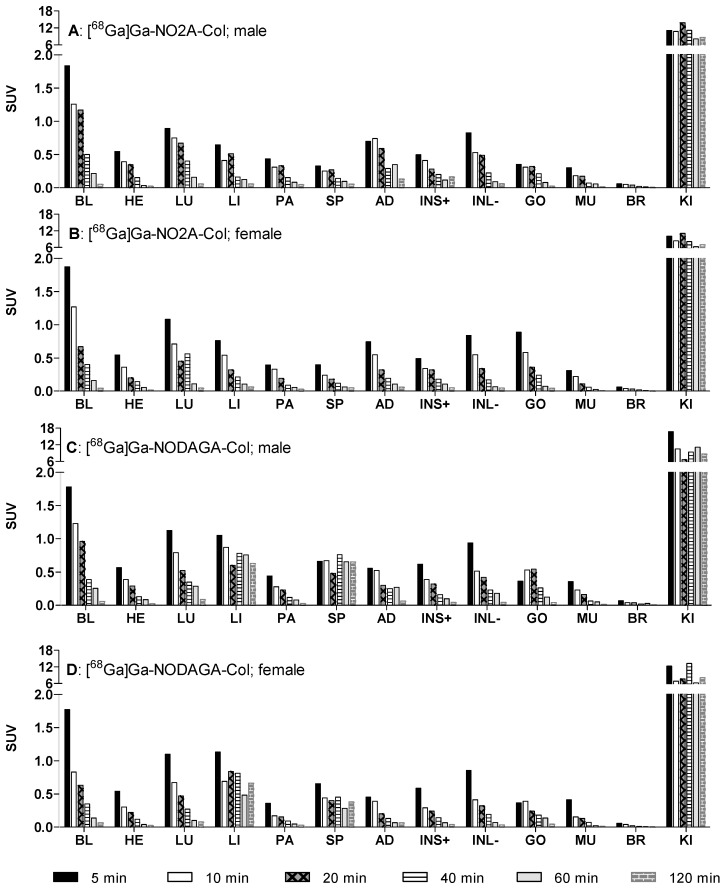
Kinetics of organ radioactivity uptake (standardized uptake value (SUV)) obtained at 5, 10, 20, 60 and 120 min after i.v. administration of [^68^Ga]Ga-NO2A-Col and [^68^Ga]Ga-NODAGA-Col in healthy male [2] and female Sprague–Dawley rats. BL: blood; HE: heart; LU: lungs; LI: liver; PA: pancreas; SP: spleen; AD: adrenals; INS+: small intestine with its content; INL: large intestine without content; UB: bladder; GO: gonads; MU: muscle; BO: bone; BM: red bone marrow; BR: brain; KI: kidneys.

**Figure 3:**
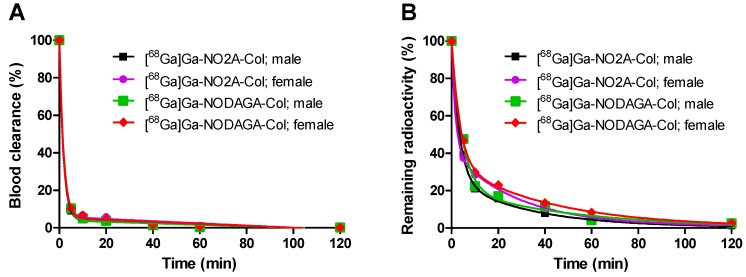
(**A**) Blood clearance of [^68^Ga]Ga-NO2A-Col and [^68^Ga]Ga-NODAGA-Col administered intravenously (R^2^ = 0.9988 (■), 0.9994 (●), R^2^ = 0.9989 (■) and R^2^ = 0.9994 (♦)) with the respective half-life values of 1.19, 1.21, 1.23 and 1.29 min. (**B**) Elimination kinetics of [^68^Ga]Ga-NO2A-Col and [^68^Ga]Ga-NODAGA-Col administered intravenously with the respective half-life values for the fast/slow phases of 19.77/1.15 min (R^2^ = 0.9994 (■)), 22.89/2.29 min (R^2^ = 0.9992 (●)), 29.33/2.41 (R^2^ = 0.9995 (■)) and 36.55/3.14 (R^2^ = 0.9958 (♦)).

Twenty four rats (12 female and 12 male; Table 1) were injected either with [^68^Ga]Ga-NO2A-Col or [^68^Ga]Ga-NODAGA-Col. The organ distribution of the tracers was assessed in 19 organs and presented as decay-corrected SUV values (Figure 2). *Ex vivo* organ distribution data revealed fast blood clearance and washout from most of the organs with SUVs below one (Figure 2 and Figure 3). The high kidney uptake (SUV > 10) indicated renal excretion both in male and female rats.

The blood clearance kinetics was best described by a single exponential phase accounting for over 98% of the radioactivity, followed by a linear phase (Figure 3A; R^2^ = 0.9988 (male, [^68^Ga]Ga-NO2A-Col); 0.9994 (female, [^68^Ga]Ga-NO2A-Col); 0.9989 (male, [^68^Ga]Ga-NODAGA-Col); 0.9994 (female, [^68^Ga]Ga-NODAGA-Col). The half-life values for the blood clearance were 1.21 min and 1.19 min, respectively, for males and males injected with [^68^Ga]Ga-NO2A-Col. The half-life values for the blood clearance were 1.23 min and 1.29 min, respectively, for males and males injected with [^68^Ga]Ga-NODAGA-Col.

The elimination was determined together with the remaining radioactivity (not excreted) for each animal and time point (5, 10, 20, 40, 60 and 120 min). The radioactivity was rapidly eliminated in both genders. The elimination kinetics was best described to be two-phase exponential with fast and slow phases. The half-life values were 1.15 and 19.77 min for males and 2.29 and 22.89 min for females injected with [^68^Ga]Ga-NO2A-Col and 2.41 and 29.33 min for males and 3.14 and 36.55 min for females injected with [^68^Ga]Ga-NODAGA-Col (Figure 3B; R^2^ = 0.9994, 0.9992, 0.9995 and 0.9958). Only 1.9% (male) and 1.4% (female) of radioactivity remained in the animals 120 min after administration of [^68^Ga]Ga-NO2A-Col. Respectively, 2.6% (male) and 2.5% (female) of radioactivity remained in the animals 120 min after administration of [^68^Ga]Ga-NODAGA-Col. The elimination kinetics was similar for the female and male animals.

The radioactivity was considerably diminished after 40 min in most other organs, except for kidneys in both genders (Figure 2). The organs that presented difference between [^68^Ga]Ga-NO2A-Col and [^68^Ga]Ga-NODAGA-Col were liver and spleen. Over 85% of the radioactivity stayed in these organs in the case of [^68^Ga]Ga-NODAGA-Col.

The reliability of the results was accessed by the goodness of the mathematical function fit to the experimental data points. This approach allows the accurate description of the process and a much lower number of sacrificed animals. It can be justified by high R^2^ values and the absence of outlying points. The main aim of the measurements was the determination of the cumulated radioactivity, which is determined by the integration of the area under the curve. Despite the gender, the pattern of distribution kinetics was similar with rapid radioactivity elimination and blood clearance. Due to the accuracy of the results, ethical considerations discouraged us from increasing the amount of animals that would be sacrificed unnecessarily in the experiment.

### 3.3. Dosimetry

**Figure 4:**
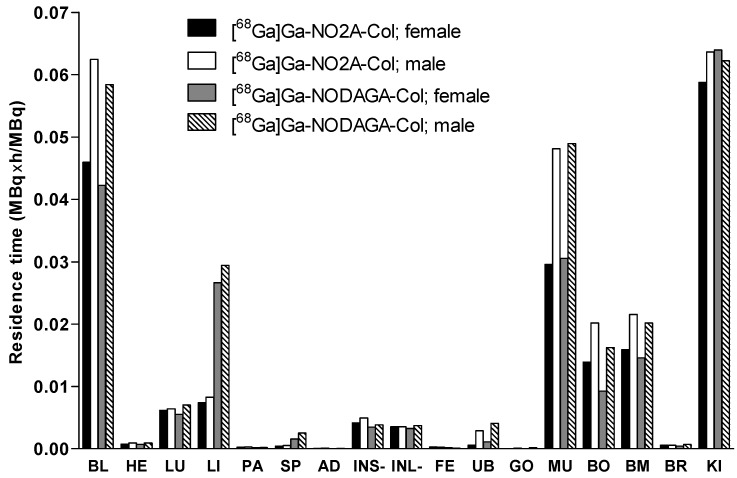
Graph showing the residence times of [^68^Ga]Ga-NO2A-Col and [^68^Ga]Ga-NODAGA-Col in different organs. The values were extrapolated to human female and male from rat *ex vivo* organ distribution data and used for dosimetric calculations using the OLINDA/EXM 1.1 software. BL: blood; HE: heart; LU: lungs; LI: liver; PA: pancreas; SP: spleen; AD: adrenals; INS-: small intestine without its content; INL-: large intestine without its content; FE: feces; UB: bladder; GO: gonads; MU: muscle; BO: bone; BM: red bone marrow; BR: brain; KI: kidneys.

The similarity of the biodistribution pattern in human and rat and the homogeneous distribution of radioactivity throughout the organ were two basic assumptions considered when extrapolating rat biodistribution data to the human species for the calculation of the absorbed and total effective doses [13]. The organ distribution study was conducted for 120 min. The residence time was determined using the trapezoidal rule, wherein the area from the injection time to the termination time was calculated. The residence time results for [^68^Ga]Ga-NO2A-Col and [^68^Ga]Ga-NODAGA-Col are summarized in Figure 4. The longest residence times were observed for the kidneys, followed by blood and muscle. The values were higher for male animals. This is because the residence time is integrated over the entire tissue using the standardized human dosimetry model of organ volumes, where in the human male model, the muscle volume is around 1.65-times higher than that in the female model (28 *vs.* 17 kg), and the male model blood volume (5.5 L) is almost 1.5 larger than in the female model (3.8 L). The other tissues are also larger in the male model, but to a lesser extent. During the calculation of the absorbed dose in each tissue (mSv/MBq) by OLINDA, the values are again divided with each tissue’s volume, providing final dose values.

Absorbed doses and total effective doses were calculated using residence time values as the input into OLINDA/EXM 1.1, the MIRD scheme of an adult male (70 kg) [12] and recommended tissue weighting factors from the International Commission on Radiological Protection (ICRP, 2007). The absorbed doses (mGy/MBq) and total effective doses (mSv/MBq) obtained from OLINDA/EXM 1.1 calculations are presented in Table 2. The extrapolated absorbed dose for both [^68^Ga]Ga-NO2A-Col and [^68^Ga]Ga-NODAGA-Col was highest in kidneys for female and male. The total effective dose was 0.0156 and 0.0162 mSv/MBq, respectively, for male and female in the case of [^68^Ga]Ga-NO2A-Col and 0.0141 and 0.0164 mSv/MBq, respectively, for male and female in the case of [^68^Ga]Ga-NODAGA-Col. The absorbed doses were slightly higher for females than for males for practically all organs.

## 4. Discussion

The practicality of the estimation of the human organ and whole-body radiation dosimetry based on animal organ biodistribution data has been demonstrated previously, e.g., for radiolabeled somatostatin [16,17,18] and exendin-4 analogues [19]. The biodistribution variation between different species, e.g., rats, mice [17,20] and human, must be considered. However, significant correlation has been found between the absorbed doses for kidneys estimated in an animal study and obtained from a clinical study [16,21,22].

The basic assumptions that are made when extrapolating the rat biodistribution data to the human species for the calculation of the absorbed and total effective doses are the similarity of the biodistribution pattern in human and rat and the homogeneous distribution of radioactivity throughout the given organ [13]. The organ distribution study was conducted for 120 min, while the theoretical residence time for ^68^Ga(III), assuming retention in the organism for its whole physical life-span, was approximately 98 min. One more conservative approximation was calculating the residence time, which was theoretically equal to the area under the organ time radioactivity curves, from time zero to infinity. In practice, the area from the injection time to the termination time was used, and it was assumed that the further decline in radioactivity occurred due to physical decay without further biological clearance. The experimental protocol covered the major part of the radioactive decay of ^68^Ga and, thus, the remaining decays after the last measurement counts to a small percentage of the total number of disintegrations in each organ. Consequently, the uncertainty in extrapolation after 120-min measurements has only minor effects on the total estimate of the time-integrated activity. As mentioned above, the biodistribution and pharmacokinetics were investigated for 120 min, exceeding the theoretical residence time of 98 min, and, thus, providing the high accuracy of the radiation-absorbed dose estimation.

SUVs continuously declined for most of the organs (Figure 2) for [^68^Ga]Ga-NO2A-Col, while SUVs in the liver and spleen were approximately constant starting at about 10 min after injection for [^68^Ga]Ga-NODAGA-Col. Accumulation in the kidneys was observed throughout the experiment duration for both agents, as was observed also previously in *in vivo* PET images [2]. The dosimetry allometrically scaled to human from animals of both genders is required for the potential translation of the imaging agents to clinical use. No difference in biodistribution pattern dependent on the gender could be observed for either [^68^Ga]Ga-NO2A-Col or [^68^Ga]Ga-NODAGA-Col for most of the organs. The slightly faster blood clearance for female rats resulted in lower residence time for blood and organs, reflecting the blood radioactivity. The relatively high blood and tissue clearance rate is expected to provide high contrast already 60 min post-injection for both [^68^Ga]Ga-NO2A-Col and [^68^Ga]Ga-NODAGA-Col. The fast blood clearance could also be observed by PET imaging, where the background radioactivity level diminished already 25 min post-injection [2]. [^68^Ga]Ga-NO2A-Col and [^68^Ga]Ga-NODAGA-Col were washed out from most of the organs, except for kidney for [^68^Ga]Ga-NO2A-Col and kidney, spleen and liver for [^68^Ga]Ga-NODAGA-Col within 2 h, presumably through renal excretion (Figure 2 and Figure 3). The uptake in most of the vital organs, including heart and lung, demonstrated SUVs below 0.5. The uptake was reduced to SUVs below 0.1 within 120 min in such organs as blood, heart, lung, pancreas, adrenal gland, small intestine, large intestine, testis, muscle and brain, thus indicating low radiation dose to those sensitive organs. The lowest uptake was observed in the brain with an SUV below 0.01 for both [^68^Ga]Ga-NO2A-Col and [^68^Ga]Ga-NODAGA-Col, most probably corresponding to the radioactivity content in the blood. The uptake in small and large intestines (SUV < 0.1) was low, with rapid washout. The low healthy organ uptake observed in the biodistribution study presumably indicated low binding of the agents to the constitutive collagen. Residence times were slightly higher for male animals for both [^68^Ga]Ga-NO2A-Col and [^68^Ga]Ga-NODAGA-Col as a consequence of the intrinsic phenomena related to the definition and calculation methodology of the residence time.

The statistical comparison of [^68^Ga]Ga-NO2A-Col and [^68^Ga]Ga-NODAGA-Col biodistribution in healthy male Sprague–Dawley rats 1 h post-injection demonstrated previously a difference in liver, spleen and kidney uptake [2]. The difference was presumably attributed to the variation in the charge of the complex moiety of the analogues. The lower uptake was found for [^68^Ga]Ga-NO2A-Col with a positively-charged complex moiety. A difference in organ absorbed doses was observed for the liver and spleen in this study, as well. The higher uptake for [^68^Ga]Ga-NODAGA-Col retained for 120 min was reflected in the longer residence time and higher absorbed dose to these organs (Figure 4, Table 2). Thus, the shorter residence time of [^68^Ga]Ga-NO2A-Col in liver and spleen as compared to that of [^68^Ga]Ga-NODAGA-Col resulted in a lower absorbed dose, making the former analogue more preferable. The values were higher for [^68^Ga]Ga-NODAGA-Col by 100% for the liver of both male and female animals and by 50% and 80% for the spleen, respectively, for the female and male animals. Nevertheless, the absorbed doses can be considered very low for both tracers, and as reported previously [2], the radioactivity uptake in the liver and spleen in the PET images was too low for the visualization, even for [^68^Ga]Ga-NODAGA-Col. In clinical application, this would provide a high contrast image, enabling the unambiguous detection of a lesion when using either of the tracers.

The dose was higher for females by 10%–30% in thyroid, skin, lung, heart wall, muscle, brain, stomach wall, liver and total body, even though the measured SUV was similar in many of these tissues. Higher calculated absorbed doses in females are likely due to the particular gender phantom model used by OLINDA, in particular the cross-fire effect from female organs, such as breasts. The absorbed dose to the reproductive organs was approximately 1.5-times higher for male animals, but there was no difference between the agents.

Radiation-sensitive organs, such as bone marrow and reproductive organs, also demonstrated favorable dosimetry. There was no difference in bone marrow absorbed dose between the female and male rats or between the agents. The absorbed dose to bone red marrow was not the restricting parameter. It was 0.0170 (female) and 0.0167 (male) mGy/MBq for [^68^Ga]Ga-NO2A-Col and 0.0161 (female) and 0.0159 (male) mGy/MBq for [^68^Ga]Ga-NODAGA-Col that yielded over 200 GBq, able to provide over 1000 PET examinations per year before reaching the red marrow limiting dose of 2 Gy [23].

The kidneys demonstrated the highest uptake, which is rather common for peptide-based tracers. It is worth mentioning that renal excretion is more preferable as compared to hepatobiliary and gastrointestinal, since it is faster and the bladder can be voided within an hour, resulting in lower cross-radiation dose from the kidney as a source organ to the healthy vital organs. Moreover, the well-defined structure of kidneys and bladder is easier to identify in the image. The domination of kidney uptake and slow excretion reflected the two-phase exponential elimination kinetics determined from organ distribution experiments. While our previous study demonstrated a slight difference, though statistically significant, in kidney uptake between the two tracers, in this study, the absorbed dose was similar (Table 2). The prolonged retention time of the radioactivity in the kidneys resulted in the high residence time of 0.062 ± 0.002 MBq × h/MBq. The absorbed dose of 0.1 mGy/MBq was the highest amongst the organs for these agents and comparable to other peptide based tracers, e.g., somatostatin analogues with absorbed dose of 0.093 mGy/MBq ([^68^Ga]Ga-DOTA-TATE; DOTA: 1,4,7,10-tetraazacyclo-dodecane-1,4,7,10-tetraacetic acid; TATE: d-Phe^1^-Tyr^3^-octreotate) and 0.082 mGy/MBq ([^68^Ga]Ga-DOTA-TOC; TOC: d-Phe^1^-Tyr^3^-octreotide) [24] or lower as compared to the exendin-4 analogue with a value of 0.276 mGy/MBq [21]. The maximum total amount of radioactivity of [^68^Ga]Ga-NO2A-Col and [^68^Ga]Ga-NODAGA-Col that could be administered to a subject, if solely restricted by the dose absorbed by kidneys, would respectively be 1456 (female)/1471 (male) MBq and 1351 (female)/1506 (male) MBq before reaching a kidney-tolerable absorbed dose of 150 mGy [25]. The absorbed dose to this organ assuming the administration of 100 MBq was about 20% ([^68^Ga]Ga-NO2A-Col) and 21% ([^68^Ga]Ga-NODAGA-Col) of the maximum allowed dose of 50 mGy to a single organ in an adult research subject [26].

The estimated whole body effective dose was similar for [^68^Ga]Ga-NO2A-Col and [^68^Ga]Ga-NODAGA-Col when extrapolated from the female rat measurements. These values were higher than those for male rat measurements (Table 2). Administration of 50–100 MBq of [^68^Ga]Ga-NO2A-Col or [^68^Ga]Ga-NODAGA-Col would yield a dose within the range of 0.78–1.64 mSv. In addition, computed tomography examination (CT), which provides morphological information and is used for attenuation correction, would result in an extra 1 mSv. Thus, the total dose per examination would correspond to 1.78–2.64 and allow for 3–6 PET-CT examinations annually before reaching the dose limit defined for healthy volunteers of not more than 10 mSv. The maximum total amount of radioactivity of [^68^Ga]Ga-NO2A-Col and [^68^Ga]Ga-NODAGA-Col that could be administered to a subject if restricted by effective dose (Table 2) would respectively be 617 (female)/641 (male) MBq and 610 (female)/709 (male) MBq before reaching the limit for healthy volunteers of 10 mSv. The low healthy organ uptake observed in the biodistribution study presumably indicated low binding of the agents to the constitutive collagen. Consequently, a lower effective dose could be expected as compared to ^68^Ga-based peptide imaging agents with the target physiologically expressed in healthy organs. For example, somatostatin receptors expressed in adrenal glands, pituitary gland and pancreas result in an effective dose of 0.021 mSv/MBq for both [^68^Ga]Ga-DOTA-TATE and [^68^Ga]Ga-DOTA-TOC. Thus, the parameter restricting the administered dose was the total effective dose.

Although the highest organ absorbed dose was found for kidneys (0.1 mGy/MBq), this organ was not the administered the dose-limiting one; while the total effective dose was the limiting parameter with 0.0162/0.0156 (female/male) mSv/MBq and 0.0164/0.0141 (female/male) mSv/MBq, respectively, for [^68^Ga]Ga-NO2A-Col and [^68^Ga]Ga-NODAGA-Col. This corresponded to the total amount of radioactivity that could be administered per year of 610–709 MBq, allowing up to six examinations per year for both [^68^Ga]Ga-NO2A-Col and [^68^Ga]Ga-NODAGA-Col. However, a two-fold lower absorbed dose for [^68^Ga]Ga-NO2A-Col in liver and spleen makes this tracer more attractive. As mentioned earlier, the dosimetry measurements consider the estimation of the absorbed doses to the healthy organs and tissue in order to determine the limit of the administered radioactivity dose. However, it should be kept in mind that the biodistribution pattern might be influenced by the disease, and thus, dosimetry monitoring should be conducted during the clinical examinations.

## 5. Conclusions

The biodistribution of [^68^Ga]Ga-NO2A-Col and [^68^Ga]Ga-NODAGA-Col in rat was investigated as a function of time, and the data were used for the calculation of radiation dosimetry in humans. The limiting parameter for the administered dose was the total effective dose that would allow for up to six examinations per year in humans without exceeding the radiation limits in critical organs. It might be sufficient for adequate disease monitoring in longitudinal studies and routine clinical setup.

## Figures and Tables

**Table 1:** The weight of the rats and injected radioactivity doses of [^68^Ga]Ga-NO2A-Col and [^68^Ga]Ga-NODAGA-Col.

Tracer	*N* (gender)	Animal Weight *, (g)	Injected Dose *, (MBq)
[^68^Ga]Ga-NO2A-Col	6 (male)	397 ± 18	8.5 ± 1.5
[^68^Ga]Ga-NO2A-Col	6 (female)	261 ± 23	7.7 ± 2.6
[^68^Ga]Ga-NODAGA-Col	6 (male)	374 ± 28	17.3 ± 1.7
[^68^Ga]Ga-NODAGA-Col	6 (female)	243 ± 7	11.9 ± 4.4

* Mean ± SD, *N* = 6.

**Table 2:** Estimated absorbed doses (mGy/MBq) of [^68^Ga]Ga-NO2A-Col or [^68^Ga]Ga-NODAGA-Col in human females and males extrapolated from rat organ distribution data.

Organ	[^68^Ga]Ga-NO2A-Col		[^68^Ga]Ga-NODAGA-Col	
	Female	Male	Female	Male
Kidneys	0.103	0.102	0.111	0.100
Adrenals	0.009	0.009	0.009	0.008
Liver	0.007	0.006	0.014	0.012
LLI wall *	0.022	0.020	0.023	0.020
ULI wall **	0.019	0.016	0.019	0.015
Red marrow	0.017	0.017	0.016	0.016
Spleen	0.008	0.006	0.011	0.011
Osteogenic cells	0.032	0.025	0.030	0.024
Small intestine	0.020	0.018	0.020	0.018
Ovaries/testes	0.009	0.015	0.009	0.015
Urinary bladder wall	0.016	0.013	0.017	0.018
Breasts	0.016	N/A ***	0.016	N/A ***
Uterus	0.019	N/A ***	0.019	N/A ***
Stomach wall	0.019	0.015	0.019	0.015
Skin	0.015	0.012	0.015	0.011
Lungs	0.009	0.007	0.008	0.007
Heart wall	0.008	0.006	0.008	0.006
Muscle	0.006	0.005	0.006	0.005
Pancreas	0.008	0.007	0.008	0.007
Brain	0.003	0.002	0.002	0.002
**Total body**	0.018	0.015	0.018	0.015
**Total effective dose (mSv/MBq)**	**0.016**	**0.016**	**0.016**	**0.014**

* LLI: lower large intestine; ** ULI: upper large intestine; *** N/A: not applicable.
